# Distinct capabilities of different Gammaproteobacterial strains on utilizing small peptides in seawater

**DOI:** 10.1038/s41598-019-57189-x

**Published:** 2020-01-16

**Authors:** Shuting Liu, Zhanfei Liu

**Affiliations:** 1 The University of Texas at Austin, Marine Science Institute, Port Aransas, TX USA; 2 Present Address: Marine Science Institute, University of California Santa Barbara, Santa Barbara, CA 93106 USA

**Keywords:** Water microbiology, Element cycles

## Abstract

Proteins and peptides account for 20–75% of marine biota biomass, of which a major fraction is metabolized by bacteria, thus deciphering interactions between bacteria and peptides is important in understanding marine carbon and nitrogen cycling. To better understand capabilities of different bacterial strains on peptide decomposition, four Gammaproteobacteria (*Pseudoalteromonas atlantica*, *Alteromonas sp*., *Marinobacterium jannaschii*, *Amphritea japonica*) were incubated in autoclaved seawater amended with tetrapeptide alanine-valine-phenylalanine-alanine (AVFA), a fragment of RuBisCO. While AVFA was decomposed greatly by *Pseudoalteromonas atlantica* and *Alteromonas sp*, it remained nearly intact in the *Marinobacterium jannaschii* and *Amphritea japonica* incubations. *Pseudoalteromonas* and *Alteromonas* decomposed AVFA mainly through extracellular hydrolysis pathway, releasing 71–85% of the AVFA as hydrolysis products to the surrounding seawater. Overall, this study showed that Gammaproteobacterial strains differ greatly in their capabilities of metabolizing peptides physiologically, providing insights into interactions of bacteria and labile organic matter in marine environments.

## Introduction

The microbial loop plays a significant role in shaping the temporal and spatial distributions and pathways of bioelements such as C, N, P, and Fe in seawater^1^. The microbial loop converts organic matter from dissolved to particulate phase through the buildup of microbial biomass that is further passed to higher trophic levels via grazing, thus the interaction between bacteria and dissolved organic matter (DOM) is one of the major factors determining carbon flux in the ocean^2^. Labile organic matter, such as proteins and peptides in either dissolved or particulate phases, turns over rapidly in seawater, supporting a major fraction of bacterial growth^3,4^. To be available for bacteria, proteins and peptides need to be first cleaved into small peptides (ca. <600 Da) outside cell membrane by extracellular hydrolysis^5^. Therefore, it is important to study decomposition efficiency and pathways of small peptides in order to understand C and N cycling rates and factors controlling the bacteria-DOM interactions in seawater^6–8^.

Knowing “who is doing what” is a fundamental question in the field of microbial ecology, and identifying biogeochemical function of different bacteria is key to understanding their ecological niche in the environment and their contribution to biological processes. Previously, differentiating the capability of different bacteria on labile DOM decomposition is mainly based on examining the change of bacterial community structures. Copiotrophic bacteria often dominate bacterial community after labile DOM is introduced to seawater incubations^9–15^. For example, Alphaproteobacteria (including *Roseobacter*), Gammaproteobacteria (including *Alteromonas*), Flavobacteria/ Sphingobacteria, Bacteroidetes, and Verrucomicrobia can outcompete other bacterial taxa and dominate bacterial community structure in incubations after specific labile compounds are added, such as peptides, proteins, amino acids and polysaccharides^16–21^. The evolving dominance of certain bacterial taxa with incubation time implies that different bacterial taxa may have different capabilities on decomposition of labile DOM, and certain copiotrophic bacteria may metabolize the substrate faster than others. Through the techniques of Microautoradiography-Fluorescent *in situ* hybridization (MAR-FISH) and stable isotope probing (SIP), it is identified that Alphaproteobacteria such as *Rhodobacterales* and *Ruegeria*, Gammaproteobacteria such as *Alteromonas*, and Flavobacterium group efficiently take up labile organic matter including peptides, proteins, amino acids or sugars in natural waters, further indicating their outstanding capability of labile substrate utilization^22–25^.

Not only the efficiency, but also the pathway of peptide decomposition may differ among bacterial populations. To date, there are two main proposed pathways of small peptide decomposition. The first pathway is that peptide is hydrolyzed into free amino acids outside the cytoplasmic membrane by extracellular enzymes, which are either dissolved freely in the water or attached to the cell wall or in the periplasmic space, and then these amino acids are metabolized^26–29^. The second pathway is that intact small peptides are directly transported into the cell via transporters, i.e., peptide permeases that are located across the cytoplasmic membrane, before being metabolized intracellularly^30,31^. Production of peptide fragments and amino acids in the surrounding water during peptide decomposition serves as evidence of the first pathway, when extracellular hydrolysis outpaces uptake of hydrolysis products^16,32,33^. In comparison, the existence of different peptide transporters targeting specific peptide substrates in bacteria, or the presence of peptide transporter genes in seawater, supports the second pathway^34–37^.

Even though a tight relationship between bacterial communities and peptide decomposition has been suggested^25^, direct evidence of whether different bacteria differ in their peptide decomposition capabilities is lacking. Protein decomposition in single bacterial strain cultures has been studied^38–40^, but small peptide decomposition has not been well-explored. The advantage of using small peptides is that the simple structure of peptides allows one to elucidate its decomposition pathways via the produced fragments and metabolites during decomposition. Comparison among different bacterial species not only in DOM decomposition rates, but also in decomposition pathways is needed to better understand their potential difference of physiological response to environmental signals.

To better understand capabilities of different bacteria on peptide decomposition, we incubated a model tetrapeptide, alanine-valine-phenylalanine-alanine (AVFA), with four individual Gammaproteobacterial strains, including *Pseudoalteromonas atlantica*, *Alteromonas sp*., *Marinobacterium jannaschii*, and *Amphritea japonica*. AVFA is a fragment of ribulose-1,5-biphosphate carboxylase/oxygenase (RuBisCO) that is ubiquitously involved in photosynthesis, and has been applied in our previous peptide decomposition studies^16,25^. These four bacterial strains were chosen because their growth has been often observed in substrate addition incubations in coastal waters^41–45^ (8-3,100 fold change of relative abundance from amplicon sequencing, Table 1). We aim to obtain direct correlations between individual bacterial strains and their peptide decomposition rates and pathways, which can offer key insights into peptide decomposition in natural seawater.

**Table 1:** Specific growth rate (μ), generation time, maximal bacterial abundance fold increase of four bacterial strains in AVFA and control (CTR) treatments during incubation in this study, and comparison with maximal % fold increase in AVFA incubations within 72 h based on DNA amplicon sequencing data from cited previous studies using natural bacterial assemblages.

Bacterial strain	In CTR treatments			In AVFA treatments			
	μ (day^−1^)	generation time (h)	max abundance fold increase in this study	μ (day^−1^)	generation time (h)	max abundance fold increase in this study	max % fold increase in other studies
*Pseudoalteromonas*	2.02	8	2.1	3.50	5	3.2	17^16^
*Alteromonas*	1.11	15	1.6	2.58	6	3.2	3100^43^
*Marinobacterium*	1.25	13	1.1	1.76	9	1.2	8^16^
*Amphritea*	5.31	3	2.8	3.90	4	2.6	>500^16,45^

Note that the fold increase of bacterial abundance in this study may not be directly comparable to that from sequencing data from other studies as uneven gene copy numbers among bacteria were amplified for sequencing, thus interpretation should be in caution.

## Results

### AVFA decomposition rates and bacterial abundance

**Figure 1:**
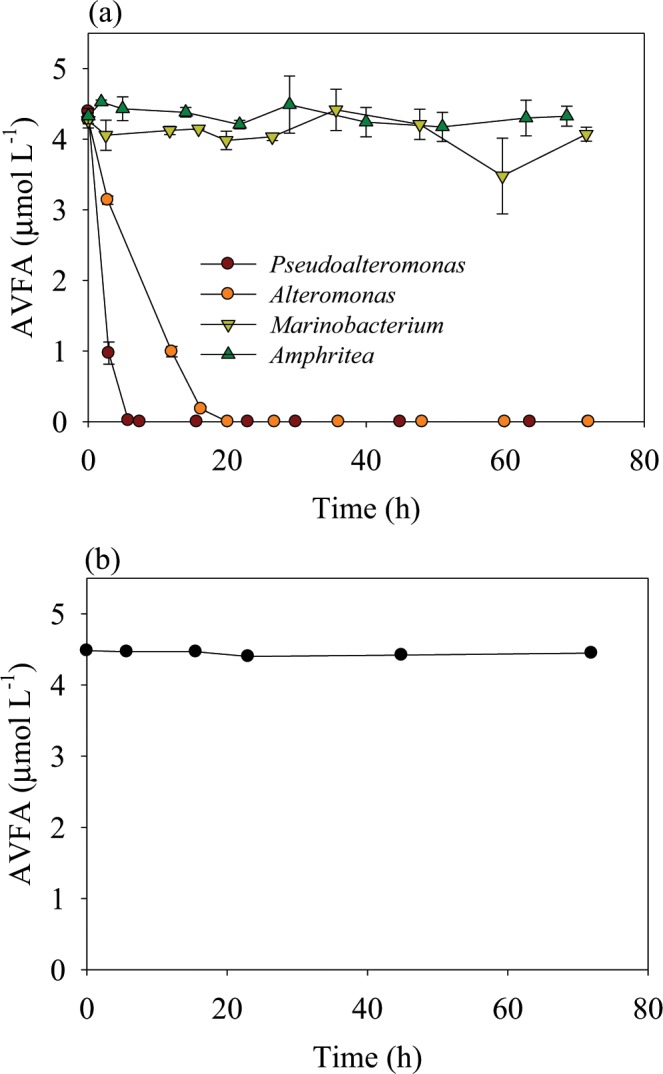
Changes of AVFA concentrations with incubation time in (**a**) four bacterial strain treatments (data points were presented as average ± standard deviation of duplicates) and (**b**) seawater without bacterial strain.

AVFA decomposition rates differed notably among the four bacterial strains during the 72 h incubation (Fig. 1a). While AVFA concentrations did not show much change in the *Marinobacterium* and *Amphritea* incubations within 72 h, they decreased to undetectable values within the initial 7–20 h in the *Pseudoalteromonas* and *Alteromonas* incubations. In contrast, AVFA concentrations remained nearly constant in the seawater control without bacteria (Fig. 1b), showing that decomposition of AVFA in the treatments was caused by the bacterial strains inoculated.

**Figure 2:**
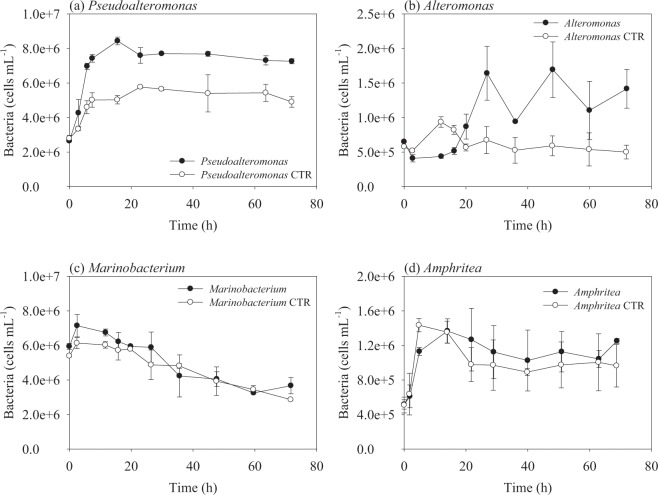
Changes of bacterial abundance with incubation time in each bacterial strain treatments and their corresponding control (CTR) treatments (without AVFA amendment). Data points were presented as average ± standard deviation of duplicates.

Corresponding to the peptide decomposition within the initial 7–20 h, bacterial abundance increased about 3 times for the *Pseudoalteromonas* and *Alteromonas* incubations (Fig. 2, Table 1). The specific growth rates were 3.50 day^−1^ (generation time = 5 h) and 2.58 day^−1^ (generation time = 6 h) for *Pseudoalteromonas* and *Alteromonas*, respectively, in the AVFA treatments, in comparison to about half specific growth rate in corresponding controls without AVFA added (Table 1). The increase of bacterial abundance in the control treatments was possibly due to bacterial utilization of natural dissolved organic matter (DOM) in the seawater medium, but this increase was only 10–40% of those treatments with AVFA added. In contrast, bacterial abundances in the *Marinobacterium* and *Amphritea* incubations remained more or less similar to, or even less than, those in their corresponding control treatments, with maximal 1.1–2.8 fold increase during incubation (Table 1).

AVFA decomposition with time by *Pseudoalteromonas* and *Alteromonas* followed exponential decay (Fig. 1), suggesting that the decomposition followed first-order reaction. The decomposition rate constants (y = a × e^−bx^, in which b represents rate constant) were obtained for each bacterial strain. Since the initial bacterial abundances varied, the rate constant of AVFA decomposition was normalized to initial bacterial abundance for comparison. The normalized rate constants differed significantly among the four bacterial strains (ANOVA p = 1.94 × 10^−8^). *Pseudoalteromonas* showed the highest decomposition rate (3.6 × 10^−7^ mL h^−1^ per cell), followed by *Alteromonas* (2.8 × 10^−7^ mL h^−1^ per cell). In contrast, there was essentially no decomposition by *Marinobacterium* and *Amphritea* (Bonferroni t test, p < 0.05).

### Peptide fragment and amino acid production from AVFA hydrolysis

**Figure 3:**
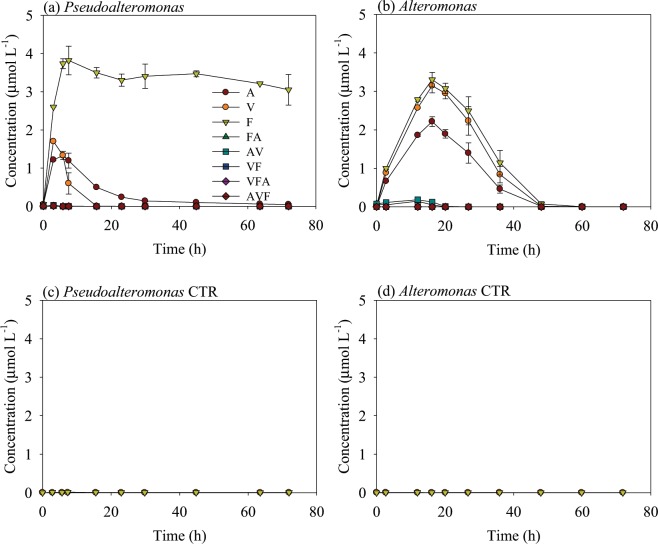
(**a**,**b**) Changes of concentrations of peptide fragments and amino acids with incubation time in the *Pseudoalteromonas* and *Alteromonas* treatments; (**c**,**d**) changes of amino acid concentrations with incubation time in the *Pseudoalteromonas* and *Alteromonas* control (no AVFA amendment) treatments. Data points were presented as average ± standard deviation of duplicates.

Concentrations of peptide fragments (FA, AV, VF, VFA and AVF) and amino acids (A, V and F) produced from AVFA hydrolysis were monitored for *Pseudoalteromonas* and *Alteromonas* (Fig. 3). Peptide fragments and amino acids accounted for 71–85% of the decomposed AVFA in the *Pseudoalteromonas* and *Alteromonas* incubations, with amino acid F concentrations reaching as high as 3.3–3.8 μmol L^−1^. The maximum fragment concentrations in these two treatments occurred at the time points when AVFA was completely decomposed (Figs. 1 and 3). Afterwards, most fragments, except F in the *Pseudoalteromonas* incubation, decreased with incubation time, indicating that these fragments were further utilized by bacteria. F in the *Pseudoalteromonas* incubation, however, remained above 3 μmol L^−1^ throughout the incubation after reaching the plateau (Fig. 3a). In all control treatments, all fragments remained at background levels (<0.009 μmol L^−1^) during the entire incubation time (Fig. 3c,d), suggesting produced peptide and amino acid fragments in the bacteria treatments were from hydrolysis of amended AVFA substrate. Since AVFA changed little during the *Marinobacterium* and *Amphritea* incubations, fragment production by these two bacteria strains was negligible and the data were not included here.

### Ammonium production

**Figure 4:**
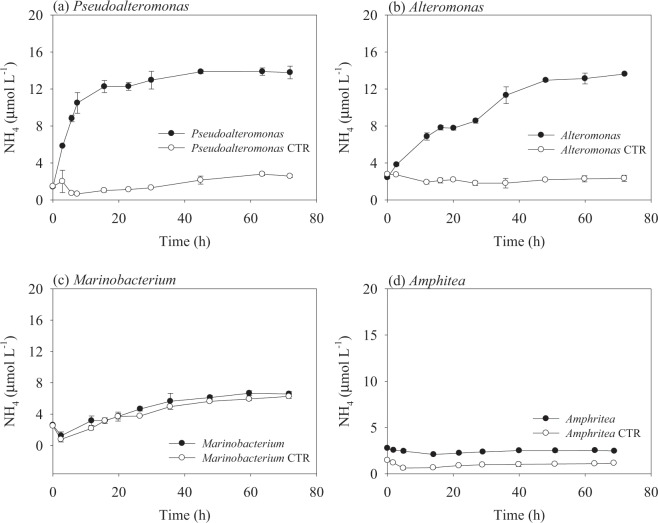
Changes of ammonium concentrations with incubation time in four bacterial strain and their corresponding control (CTR) treatments. Data points were presented as average ± standard deviation of duplicates.

As one major metabolite of peptide decomposition, ammonium was monitored in all incubations. The two bacterial treatments in which AVFA showed great decomposition, i.e., *Pseudoalteromonas* and *Alteromonas*, released 5.9–9.9 μmol L^−1^ more ammonium than their corresponding control treatments at the time point when AVFA concentrations decreased to nearly zero (Fig. 4). Whereas in the *Marinobacterium* and *Amphritea* treatments, the ammonium levels remained similar to the controls.

## Discussion

### Peptide decomposition efficiency differed greatly among the bacterial strains

Peptide decomposition rates differed greatly among the four bacterial strains tested (Fig. 1). This difference may be related to their ecological strategies for substrate utilization. Some bacteria, referred to as generalists, can use a variety of organic substrate^46–48^. In contrast, bacterial taxa that utilize specific or a limited range of organic substrates are defined as specialists. For example, the Cytophaga-Flavobacter-Bacteroidetes cluster specializes in the use of high-molecular-weight polymers including chitin and protein^10,49,50^. Gammaproteobacteria consist of diverse groups of specialized bacteria^51^. The rapid peptide decomposition by *Pseudoalteromonas* and *Alteromonas* in our data suggests that these two bacterial strains may be specialized in peptide decomposition. Generation times for *Pseudoalteromonas* and *Alteromonas* in our study ranged from 5–15 h, close to those reported for copiotrophic bacteria (9–12 h)^19^. However, not all Gammaproteobacteria utilize peptides in a same way, as *Marinobacterium* and *Amphritea* did not utilize the peptide at all. Belonging to Gammaproteobacteria, *Marinobacterium* is an aerobic helical bacteria and *Amphritea* is isolated from sediment^52–54^. It has been shown that *Marinobacterium jannaschii* can utilize amino acids but is not capable of producing gelatinase, a hydrolytic enzyme to break down proteins and peptides in gelatin^52^. *Amphritea japonica* does not produce gelatinase and protease, either^54^. Consistently, our study demonstrates that *Marinobacterium* and *Amphritea* do not utilize small peptides in marine environments, possibly due to the lack of production of peptidases to hydrolyze peptides. Their growth in previous peptide incubations using natural bacterial assemblage^16^, therefore, indicates that they may have utilized amino acids released from peptide hydrolysis by other bacteria such as *Alteromonas*. The different peptide decomposition patterns among these four Gammaproteobacteria are indicative of DOM resource partitioning among different bacteria taxa^22,55–57^. Consistently, other studies also demonstrated that hydrolysis or decomposition of labile DOM is usually controlled by specific bacterial phylotypes^15,17,18^.

Our results clearly show the distinctly different capacities of peptide utilization among the bacterial strains tested. While the current development of culture-independent techniques such as metagenomics and metatrascriptomics have advanced our knowledge of microbial ecology to a new stage, it should not detract from the attention of using model isolates to gain insights into environmental microbiomes^58^. Culture studies of single bacterial strains can particularly provide detailed physiological features of specific bacteria, an important angle to understand bacterial consortium as a whole. For example, peptide decomposition pathways, which will be discussed in the following section, can be identified clearly for individual bacterial strains.

### Peptide decomposition pathways by *Pseudoalteromonas* and *Alteromonas*

**Figure 5:**
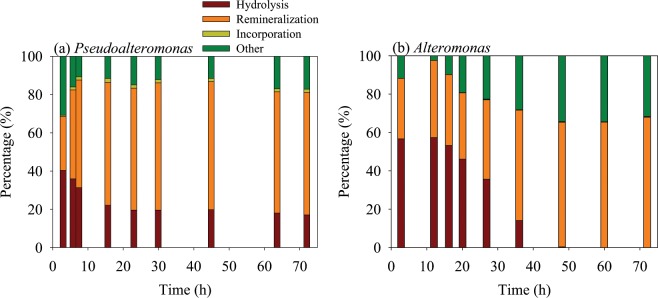
Mass balance of AVFA decomposition (including percentages of decreased peptide due to hydrolysis to fragments, remineralization to ammonium, incorporation into bacterial biomass and other unaccounted transformation) in the *Pseudoalteromonas* and *Alteromonas* treatments.

A mass balance based on nitrogen in peptide is helpful in identifying peptide decomposition pathways. One advantage of using small peptides such as AVFA is that all hydrolyzed fragments can be identified quantitatively to calculate mass balance and further derive decomposition pathways. AVFA can be hydrolyzed to peptide fragments and amino acids, remineralized to inorganic nutrients (i.e., ammonium), incorporated into bacterial biomass, and transformed to other forms^16^. We calculated the percentage of hydrolysis using the sum of hydrolysis products containing F (i.e., VFA, AVF, VF, FA, F), estimated the remineralization percentage using ammonium concentrations, calculated the nitrogen being combined into bacterial biomass assuming 20 fg C per cell and a C/N ratio of 4^59^, and estimated other forms based on mass balance (Fig. 5). The hydrolyzed fragments accounted for 31–57% of AVFA nitrogen in the *Pseudoalteromonas* and *Alteromonas* treatments before AVFA concentrations decreased to zero during the incubation, and the hydrolysis percentage decreased afterwards. This pattern is also reflected in the accumulation of high concentrations of hydrolysis products in the *Pseudoalteromonas* and *Alteromonas* incubations, accounting for up to 71–85% of the amount of AVFA lost (Fig. 3). This result indicates that the major decomposition pathway of peptide by *Pseudoalteromonas* and *Alteromonas* was extracellular hydrolysis.

Extracellular hydrolysis of peptides is either by free enzymes dissolved in the medium or through cell-associated enzymes that are attached to cell surface or in the periplasmic space^28^. Free enzymes are often operationally defined as enzymes passed through 0.2 μm filters. Contributions of free enzymes to total peptide hydrolysis vary from 10–30% to as high as 65–100% among different marine environments^60–63^, indicating only certain bacteria may possess this strategy and their presence may be dependent on the environment. Alternatively, if AVFA is hydrolyzed within the periplasmic space, the diffusion rate of AVFA hydrolysates must be higher than the uptake rate of AVFA and its hydrolysates in order to see the accumulation of amino acids in the surrounding water (Fig. 3). Diffusion of peptides and its hydrolysates is through “porin” proteins in the outer cell membrane that allow transportation of molecules smaller than ca. 600 Da^64^. Since AVFA has a molecular weight of 406 Da, AVFA and its hydrolysates can freely diffuse out of the cell via the “porin”. Diffusivity (D_f_) of hydrolysates with molecular weight of 10–10^4^ Da ranges from 1 × 10^−6^ − 2 × 10^−5^ cm^−2^ s^−1^ ^65,66^. A typical bacterial cell has a radius (r) of 0.2–0.6 μm if assuming bacteria are in spherical shape^67^. The periplasmic space spans a distance (w) of around 16 nm^68^. Assuming diffusion occurs across the whole periplasmic space cross-section area, diffuse rate (D) will be D = D_f_(πr^2^ − π(r − w)^2^) = 1.9 × 10^−14^ − 1.2 × 10^−12^ s^−1^. Based on the decomposition rate of AVFA by *Pseudoalteromonas* and *Alteromonas* in this study and previous data on amino acid uptake rate by bacteria^16^, uptake rate (U) was in the range of 4.0 × 10^−6^ – 6.2 × 10^−5^ s^−1^. Since U ≫ D, diffusion of hydrolysates from cell to media is limited, thus extracellular hydrolysis by *Alteromonas* and *Pseudoalteromonas* was more likely through free enzymes or cell surface-attached enzymes.

*Alteromonas* is a ubiquitous Gammaproteobacterium in seawater and contributes significantly to the utilization of labile DOM pool^69–73^. It often accounts for a major fraction of active bacterial community during and after phytoplankton bloom^74^. The release of several extracellular aminopeptidases and endopeptidases from the *Alteromonas* B-207 strain has been detected^75^. Consistently, high concentrations of hydrolysis products were produced in the incubation medium when AVFA was rapidly decomposed in the first 20 h (Fig. 3b). *Pseudoalteromonas* is a genus that is evolved from *Alteromonas*. Similar to *Alteromonas*, it can outcompete other species with high growth rate when high concentration of organic matter is available, such as during jellyfish bloom^76,77^. Like many other *Pseudoalteromonas*, *Pseudoalteromonas atlantica* is often found to be associated with eukaryote hosts, such as algae, or sinking particles in seawater^78–80^. They are capable of producing extracellular hydrolytic enzymes for decomposing polysaccharide and proteins^81–83^, which explains why extracellular hydrolysis dominated their decomposition pathway of peptides (Figs. 3a and 5a). When *Alteromonas* and *Pseudoalteromonas* are present in the environment, hydrolysis by free enzymes produced by them can provide substrates foraging microbial “cheaters” that cannot directly metabolize the substrates^84^, thus promoting diverse bacterial community. For example, SAR11 typically do not produce hydrolytic enzymes for large molecules, but they can cross-feed on small hydrolyzed products from other coexisting bacteria^85^.

In the *Pseudoalteromonas* and *Alteromonas* incubations, amino acids, rather than peptide fragments, were the major hydrolysis products, suggesting that extracellular enzymes produced by *Pseudoalteromonas* and *Alteromonas* hydrolyzed AVFA completely to amino acids. This pattern indicates that either AVFA was hydrolyzed stepwise by exopeptidases (targeting terminal end of peptides) rapidly or it was hydrolyzed by both exopeptidases and endopeptidases simultaneously at a similar speed. Interestingly, concentrations of released amino acids in incubation medium did not follow stoichiometry of the peptide. For instance, concentrations of A should be the highest if following the stoichiometry as there are two As in AVFA, but they were in fact lower than the concentrations of F in the *Pseudoalteromonas* and *Alteromonas* treatments. To explain the unbalanced amino acid concentrations, one possibility is that bacteria may have taken up the produced amino acids during AVFA hydrolysis. Bacteria may preferentially take up A compared to other amino acids, as a previous study showed that uptake of A by bacteria was 1.25 times as fast as that of F in coastal seawater^16^. Another possibility is that amino acids were oxidized extracellularly by oxidases, and the oxidation rates may differ among different amino acids. Cell-surface oxidation of amino acids, which generates ammonium and hydrogen peroxide, has been found in phytoplankton species and bacteria-sized organisms, especially in low-ammonium environments or with addition of amino acids^86–88^. *Pseudoalteromonas* can synthesize amino acid oxidases^89,90^, thus these enzymes can potentially be released to the solution. Oxidation of amino acids by extracellular oxidases may be energetically beneficial compared to uptake of amino acids, as it does not require synthesis of various transporters targeting different amino acids^91^. Note that amino acid F remained at a high level after being produced in the *Pseudoalteromonas* incubation, but decreased rapidly in the *Alteromonas* incubation (Fig. 3). The transport system for amino acid F is stereospecific and independent, and different bacteria may require different amount of organic nutrition for growth^92^, which may contribute to the different efficiency of metabolizing F between *Alteromonas* and *Pseudoalteromonas*. Alternatively, activities of amino acid oxidases may differ between *Pseudoalteromonas* and *Alteromonas*, leading to this difference. More work is needed to elucidate this interesting pattern.

AVFA decomposition did not contribute much to the buildup of bacterial biomass, as incorporation of peptide N into biomass only accounted for less than 2% of decomposed AVFA based on the mass balance (Fig. 5). In comparison, 30–70% of decomposed AVFA were remineralized to ammonium and this percentage generally increased with incubation time, suggesting that most peptides were utilized as an energy source rather than biomass nutrition and more hydrolyzed fragments were remineralized with incubation time. It is noted that 2–35% of decomposed AVFA were unaccounted for in addition to the hydrolysis, incorporation, and remineralization proportions. We hypothesize that this unaccounted fraction of peptides might have been transformed to other DON forms, such as recalcitrant DON that are outside of our analytical window^93–95^. For example, AVFA can be oxidized by cell-surface enzymes^87^, but more work is needed to test this possibility.

## Conclusions and Implication

Through comparing peptide decomposition by different bacterial strains, we showed that bacteria differed greatly in peptide decomposition capabilities. Our results provide direct evidence of the distinct ecological roles and physiology among different bacterial strains in terms of their capability on decomposing peptides. As there is ecological difference among bacterial strains even within the same species^96^, we used four Gammaproteobacterial strains as models for their corresponding bacterial species to demonstrate their potential capability of peptide decomposition. The rapid responses of these bacterial strains (*Pseudoalteromonas* and *Alteromonas*) to peptides are consistent to field studies of peptide decomposition using natural bacterial assemblages containing these species^16,25,43^, indicating our chosen bacterial strains can represent in some degree their corresponding species in peptide decomposition. In contrast, while *Marinobacterium* and *Amphritea* showed rapid increase in natural assemblages when incubated with AVFA^16,45^, they did not grow as single strain incubations, indicating they were using byproducts from AVFA decomposition by other bacteria in natural assemblages. Our study using single bacterial strain helps identify individual roles of different bacteria in peptide decomposition. Furthermore, our culture-based studies of bacterial isolates can be integrated to culture-independent analysis, such as metagenomics and metatranscriptomics, to better understand the ecological roles, physiology, functionality of different bacteria in the seawater. Future work involving peptide incubations in other bacterial strains such as Alphaproteobacteria will be followed to compare peptide decomposition pathways between different bacterial classes. Further study using bacterial consortium with mixture of bacterial strains can gain insight into the synergistic or antagonistic role of interactions between bacterial strains in peptide degradation.

Using small peptides as a proxy, we quantified the hydrolyzed fragments and identified peptide decomposition pathways. *Pseudoalteromonas* and *Alteromonas* utilized extracellular hydrolysis as the major decomposition pathway and released fragments into the surrounding environment, thus potentially benefitting other bacteria that can utilize the free amino acids. These data have important implications for understanding potential bacterial interspecies interactions, the factors shaping bacterial community structures in the environments, and their specific ecological roles in carbon and nitrogen cycling.

## Experimental Procedures

### Bacterial strain culturing

Four Gammaproteobacterial strains were chosen for this study based on previous studies, including *Pseudoalteromonas atlantica* T2a (ATCC 43666), *Alteromonas sp*. B-207 (ATCC 33524), *Marinobacterium jannaschii* 207 (ATCC 27135), and *Amphritea japonica* JAMM 1866 (ATCC BAA-1530)^11,16,43^. Bacterial strain pellets were activated in 50 mL autoclaved Difco marine broth 2216 (BD) at 30 °C (for *Pseudoalteromonas*, *Altermonas*, *Marinobacterium*) in an incubator shaker with the speed of 120 rpm or at 20 °C (for *Amphritea*) for 24–45 h. Bacterial growth was checked occasionally during the activation. 1 mL culture was collected and fixed with formaldehyde at a final concentration of 3% and counted using a flow cytometer. When the activated bacterial culture reached the abundance of more than 10^7^ cells/mL, 5–10 mL activated culture was then subcultured in 120–125 mL marine broth, and incubated at the same temperature for another 2–23 h. During culturing, bacterial abundance was checked frequently (every hour to a few hours) using a flow cytometer. The culturing was stopped when the bacterial growth reached the late-exponential to stationary stage.

All culture transfer operations were conducted in a 70% ethanol cleaned and UV sterilized hood. To make sure cultures were not contaminated by other non-target bacteria and only single colony was formed in each culture, culture aliquots were occasionally sampled and examined under microscope after being stained with SYBR Green. Controls with only marine broth media and no bacteria strains were also included. While bacterial cultures showed opaque color due to formation of dense bacteria colonies, controls were always clear. Each bacterial culture contained single-shape cells, indicating no contaminations.

### Peptide incubation

The bacterial culture (40 mL) was cleaned with autoclaved seawater medium to remove concentrated nutrients in marine broth medium. Autoclaved seawater medium instead of artificial seawater was chosen here to mimic natural seawater environments and ensure viable bacterial growth with a full suite of nutrients and trace elements. To prepare the seawater medium, seawater was collected from the Port Aransas ship channel (27.84°N, 97.05°W) which is connected to the western Gulf of Mexico, filtered through 0.2 μm Nylon filter (Whatman, dia. 47 mm), and autoclaved at 121 °C. The 40 mL bacterial culture was centrifuged at 4,000 rpm (×2,808 g) for 15 min, then the supernatant was discarded, and 20 mL autoclaved seawater medium was added to the leftover bacteria pellet, vortexed and centrifuged at 4,000 rpm for 15 min. The supernatant was discarded and this cleaning step with 20 mL seawater medium was repeated again. This cleaning step was used to avoid introduction of excessive DOM and inorganic nutrients from culture media to the following peptide incubations. It is possible that a small fraction of bacterial cells might have been affected in terms of its biological activity. However, we kept centrifugation force <5,000 × g to resemble unmanipulated organisms as >85% of bacteria has been tested to be viable with wash and centrifuge at 5,000 × g three times^97^. After the cleaning, 1 mL seawater medium was pipetted to dissolve the bacteria pellet and then the final volume was brought up to original 40 mL with the seawater medium. This cleaned bacterial strain culture was counted under a flow cytometer to determine original bacterial abundance for peptide incubation.

AVFA contains aromatic amino acid phenylalanine that can be detected by UV absorbance and alanine is one of the most abundant amino acids in the natural seawater^98^. AVFA was incubated with each of the four single bacterial strains. For every bacterial strain, bacterial cultures were inoculated into 20 mL autoclaved seawater medium with AVFA, and the corresponding control (CTR) treatment was bacterial cultures inoculated into 20 mL autoclaved seawater medium without AVFA. In addition, 20 mL autoclaved seawater medium with AVFA amended but without bacterial strain was included. Duplicates were included for each treatment. The initial bacterial abundance was inoculated to be ca. 5 × 10^5^ cells/mL that is close to the natural abundance in the ship channel^43^. To ensure no bacteria contamination was introduced from peptide amendment, AVFA stock solution was filtered twice through 0.2 μm sterile polyvinylidene difluoride (PVDF) filters (Whatman, dia. 13 mm) before being added to the incubation bottles and the final concentrations of AVFA were ca. 5 μmol L^−1^. All pipetting or transfer operations were conducted in a sterilized clean hood. Incubations were conducted in a series of 30 mL amber bottles at room temperature (24 °C) under dark for 72 h. At different time intervals during incubation, 1 mL aliquots were siphoned out and fixed with formaldehyde (final concentration of 3%), put in 4 °C fridge for 1–4 h, flash frozen in liquid nitrogen and then preserved at −80 °C until bacterial abundance analysis. 3 mL aliquots were filtered through 0.2 μm sterile PVDF filters for peptide and amino acid analysis and these samples were stored at −20 °C. Another 10 mL aliquots were filtered through 0.2 μm sterile PVDF filters and preserved at −20 °C for ammonium analysis.

### Bacterial enumeration and chemical analyses

Bacterial abundance was enumerated in a flow cytometer (BD Accuri C6) under blue light excitation at 488 nm after bacteria cells were stained with SYBR Green (Molecular Probes, 1:100 v/v)^16,99^. Bacteria were counted in a fixed volume mode and bacterial abundance was determined from a log-scale plot of side scattered (SSC-H) versus green fluorescence signal (FL1-H) with CFlow Plus software. Bacterial specific growth rate was calculated for exponential increase period from the growth curve (bacterial abundance vs. incubation time) based on the equation: X = X_0_e^μt^, where X_0_ is the initial bacterial abundance, μ is specific growth rate, t is time, and X is bacterial abundance at time t^100^.

Concentrations of AVFA and its hydrolyzed peptide fragments (AVF, VFA, FA, VF) were analyzed using high performance liquid chromatography (HPLC, Shimadzu Prominence) equipped with a photodiode array (PDA) detector following the established protocol^16,41^. In brief, peptides were eluted on a C_18_ column (Phenomenex Luna, 5 μm 250 × 4.6 mm) through a gradient program at a flow rate of 1 mL/min. Mobile phases consisted of solvent A as 0.05 M NaH_2_PO_4_ (pH 4.5) and solvent B as methanol. Quantification was based on external standards and absorbance at 206 nm. Duplicate sample analyses generally agreed within 5% (relative standard deviation). Amino acids and AV were measured in HPLC with fluorescence detection after pre-column *o*-phthaldialdehyde (OPA) derivatization^16,101^. Standard deviations of duplicate amino acid sample analyses were within 10–20%. Ammonium concentrations were quantified in HPLC after post-column OPA derivatization following Gardner and Stjohn^102^.
